# Macrophage polarization is associated with postoperative seroma development in breast cancer in the SerMa pilot cohort

**DOI:** 10.1038/s41598-025-17139-2

**Published:** 2025-09-12

**Authors:** Felicitas Magdalena Schneider, Nicole Pochert, Fritzi Schittek, Melitta Beatrice Köpke, Carl Mathis Wild, Johanna Marie Veh, Natalie Renate Rohrmoser, Christina Kuhn, Birgit Urban, Klaus-Henning Kahl, Mariella Schneider, Angelika Mattmer, Ludwig Christian Hinske, Regina Fluhrer, Monika M. Golas, Michael Untch, Thorsten Kuehn, Maggie Banys-Paluchowski, Udo Jeschke, Claudia Traidl-Hoffmann, Christian Dannecker, Nina Ditsch

**Affiliations:** 1 Gynecology, Obstetrics and Senology, Faculty of Medicine, University of Augsburg, Stenglinstraße 2, 86156 Augsburg, Germany; 2 Helmholtz Center Munich, Institute of Environmental Medicine, German Research Center for Environmental Health, Neuherberg, Germany; 3 National Center for Tumor Diseases (NCT), NCT WERA, Würzburg, Germany; 4 Bavarian Center for Cancer Research (BKFZ), Partner Site Augsburg, Augsburg, Germany; 5 Digital Medicine, University Hospital of Augsburg, Stenglinstraße 2, 86156 Augsburg, Germany; 6 Radiotherapy, Faculty of Medicine, University of Augsburg, Stenglinstraße 2, 86156 Augsburg, Germany; 7 Department of Anesthesiology, LMU University Hospital, LMU Munich, Munich, Germany; 8 Biochemistry and Molecular Biology, Institute of Theoretical Medicine, Faculty of Medicine, University of Augsburg, Universitätsstrasse 2, 86159 Augsburg, Germany; 9 Center for Interdisciplinary Health Research, University of Augsburg, 86135 Augsburg, Germany; 10 Centre for Advanced Analytics and Predictive Sciences (CAAPS), University of Augsburg, Universitätsstraße 12a, 86159 Augsburg, Germany; 11 Human Genetics, Faculty of Medicine, University of Augsburg, Stenglinstraße 2, 86156 Augsburg, Germany; 12 Department of Gynecology and Obstetrics, Breast Cancer Center, Helios Klinikum Berlin Buch, Berlin, Germany; 13 Department of Gynecology and Obstetrics, University Hospital Ulm, Ulm, Germany; 14 Department of Gynecology and Obstetrics, University Hospital Schleswig-Holstein, Lübeck, Germany; 15 Faculty of Medicine, Institute of Environmental Medicine and Integrative Health, University of Augsburg, Augsburg, Germany

**Keywords:** Seroma, Mastectomy, Macrophages, CD68, CD163, PPARγ, Tumor microenvironment, Cancer, Immunology, Medical research, Oncology

## Abstract

Seroma formation is a frequent complication following mastectomy in the surgical treatment of breast cancer with profound consequences for the patients including possibly quality of life impairing implant complications. The pathogenesis remains unknown, leading to a lack of efficient preventive and curative strategies. The study’s objective was to determine whether the macrophage infiltration of the tumor microenvironment and surrounding adipose tissue at the time of primary surgery is associated with postoperative seroma development. The observational monocentric SerMa pilot study was conducted from 12/2019 to 12/2022. We included 91 breast cancer and 9 carcinoma in situ cases treated with mastectomy at the University Hospital Augsburg, Germany. Patients with previous malignancies, metastatic disease and known immunodeficiency were excluded. The patients underwent different mastectomy procedures with or without implant- or expander-based breast reconstruction. The study’s main outcome was seroma formation up to six months post-surgery, determined by clinical examination and fine needle aspiration of the seroma fluid if clinically necessary. Macrophage markers (CD68 and CD163) were immunohistochemically determined in formalin fixed paraffin-embedded slides containing the primary tumor and surrounding adipose tissue. Two groups were then formed as independent variables: cases with (seroma +) and without postoperative seroma formation (seroma-). Since all parameters in this study were not normally distributed, the non-parametric Mann–Whitney-U-test was used. A p-value < 0.05 was considered statistically significant. CD68 + cells (cases with seroma (seroma +): median = 90.7 cells, IQR = 62.5–130.5; cases without seroma (seroma-): median = 64.3 cells, IQR = 47.0–115-0, p = 0.036) and CD163 + cells (seroma + : median = 58.3 cells, IQR = 33.0–91.4; seroma-: median = 40.7 cells, IQR = 28.3–55.3, p = 0.027) in the tumor microenvironment and in the surrounding adipose tissue (CD68 + cells (seroma + : median = 8.0 cells, IQR = 5.3–11.0; seroma-: median = 4.7 cells, IQR = 3.0–10.0, p = 0.013), CD163 + cells (seroma + : median = 11.0 cells, IQR = 6.7–15.0; seroma-: median = 6.7 cells, IQR = 3.0–9.7, p = 0.016)) were significantly higher in cases with postoperative seroma formation compared to cases without. In the SerMa pilot study macrophage polarization within the primary tumor and surrounding adipose tissue was associated with post-operative seroma formation in breast cancer patients. This might be a suitable biomarker for predicting a higher risk of seroma formation.

## Introduction

Seroma formation is one of the most frequent complications following mastectomy with incidences as high as 90% ^1^. They can cause substantial patient discomfort, prolongation of hospital stay, delay in wound healing and timely initiation of proper adjuvant locoregional and systemic therapy ^2^. The poor understanding concerning the exact definition ^2^ and underlying mechanisms responsible for seroma formation explain the lack of efficient prevention and therapy strategies. A seroma is generally defined as an accumulation of serous fluid ^3^, the origins of which have not yet been determined ^4^. Due to its protein concentration and cellular composition of mostly lymphocytes, it has been proposed that seroma fluid (SFl) derives from an accumulation of lymphatic fluid ^5^. However, it has been shown that seromas contain different cells, an increased amount of protein and no fibrinogen, making coagulation impossible ^6^. Due to these inconsistencies, other researchers support the hypothesis of SFl being an exudate resulting from an acute inflammatory reaction ^7,8^. Multiple risk factors for seroma development are already described in literature ^9–11^. Our published results of the SerMa pilot study reinforce the theory that seroma formation is an immunological event ^9,12,13^. Moreover, we found earlier that tumor associated macrophages (TAM) in the tumor microenvironment (TME) and surrounding adipose tissue (SAT) are negatively associated with overall survival in breast cancer ^14^. CD68 is a commonly used macrophage marker, whereas CD163 is one of several markers identifying M2 macrophages ^15^. Due to their immunomodulatory effects, macrophages are crucial for wound healing ^16^. In addition, peroxisome proliferator-activated receptor gamma (PPAR-γ) polarizes macrophages to TAM and plays a key role in suppressing scarring and/or chronic wounds ^17^.

In accordance, it has recently been proven that CD68 + positive cells are present in post-mastectomy seroma beds ^18^ and, alongside CD163 + positive cells, contribute significantly to foreign body reaction ^19,20^. In addition, soluble CD163 (sCD163) can be measured by a ELISA and has prognostic value in different cancer forms, whereas this is not possible for other soluble macrophages markers ^21^.

## Methods

### Aim, design and setting

The aim of the monocentric SerMa pilot study was to identify immune markers in tissue and blood that could be related to the development of a secondary postoperative seroma already at the time of the primary breast surgery knowing that immunological factors can be detected in the content of seromas already been described by our study group in preliminary work. Firstly, Formalin-fixed paraffin embedded (FFPE) tissue of patients with breast cancer or ductal carcinoma in situ (DCIS) was used. Secondarily the seroma fluid was analyzed of its macrophage content as comparison to the tissue composition. Thirdly, preoperative sampled blood sera analyzed for sCD163 as a possible alternative seroma prediction tool.

### Patient cohort

91 primary breast cancer and 9 carcinoma in situ (CIS) cases were included into the patient cohort of the monocentric SerMa pilot study conducted between December 2019 and December 2022 at the University Hospital Augsburg, Germany. In case of bilateral disease (7 patients), both sides were considered as separate cases. All patients were treated according to German guidelines and had undergone mastectomy without reconstruction or a skin-sparing/nipple-sparing mastectomy, with implant- or expander-based breast reconstruction. 53 cases received neoadjuvant chemotherapy prior to mastectomy. In all cases fulfilling the criteria for axillary intervention, the intervention was performed according to national and international guidelines ^22^. A total of 48 patient cases underwent sentinel lymph node biopsy (SLNB), 25 axillary lymph node dissection (ALND), 8 targeted axillary lymph node dissection (TAD), 8 SLNB + ALND and 2 TAD + ALND. An implant or expander was used in 33 cases. Patients with previous malignancies and with metastatic disease at the time of diagnosis were excluded. Patients with known immunodeficiency were excluded to avoid selection bias due to possible preexisting immunological changes in the breast tissue. All patients have given informed consent to participate, and the study protocol was approved by the ethics committee of the Ludwig-Maximilians University, Munich, Germany (03/21). The study was carried out according to the guidelines of the 1975 Declaration of Helsinki. All methods were performed in accordance with the relevant guidelines and regulations.

Out of 100 cases, 80 were eligible for the immunohistochemical analysis in the tumor microenvironment at time of surgery. Reasons for drop-out were a postoperative tumor stage of ypT0 after neoadjuvant therapy, making it impossible to evaluate the tumor and its environment (16 cases), or logistical reasons (4 cases). Out of those 80, 66 comprised enough adipose tissue for the analysis of the surrounding adipose tissue. Within the study cohort, 40 developed a seroma and were punctured one or more times. Cells of 28 SFl samples were then available for flow cytometry analysis and 49 serum samples drawn at the time of the surgery were used for analysis of sCD163. An overview of the excluded cases is provided in the supplement (Additional file 1, Additional Table 1). Four representative cases were chosen for the triple immunofluorescence analysis of CD68, CD163 and PPARγ to identify the macrophage polarization status.

### Immunohistochemical staining and evaluation of the FFPE tissues

Sections from FFPE tissue were analyzed. Macrophages (CD68 + cells) were stained using an anti-human CD68 antibody (Monoclonal Rabbit IgG, Dilution 1:1000, Cell Signaling). The CD163 antigen was stained using an anti-human CD163 antibody (Monoclonal Mouse IgG1, Dilution 1:2000, Abcam). The staining protocol and detailed information regarding the antibodies is shown in the supplement (Additional file 2, Additional Table 2).

To determine the quantity of positive cells on each slide, the number of stained macrophages in the TME and in the SAT with a distance of 500 μm from the tumor stroma was counted in three representative areas at a magnification of 40 × lens. The average amount of positive cells on each slide was calculated and used for the statistical analyses.

The evaluation was performed by two independent observers. In instances of discordance between the evaluations of the two observers (eight samples, n = 2.7%), the affected cases were assessed together. This joint assessment allowed both observers to reach a consensus on the results.

### Triple immunofluorescence staining (CD68, CD163, PPARγ)

Triple immunofluorescence staining was used to evaluate the simultaneous expression of CD68, CD163 and PPARγ in the TME and SAT. The macrophage polarization status was identified on representative FFPE slides, regardless of postoperative seroma formation. Anti-human CD68 (Monoclonal Rabbit IgG, Dilution 1:1000, Cell Signaling), anti-human CD163 (Monoclonal Mouse IgG1, Dilution 1:2000, Abcam) and anti-human PPARγ (Polyclonal Rabbit IgG, Dilution 1:100, Abcam) antibodies were used as primary antibodies. The secondary antibodies resulted in a red staining of CD68 + antigens, a violet staining of CD163 + antigens and a green staining of PPARγ. Cell nuclei were stained blue using DAPI (4′,6-diamidino-2-phenylindole).

The staining protocol and detailed information regarding antibodies and fluorochromes are provided in the supplement (Additional file 3, Additional Table 3). The triple staining was evaluated using a fluorescence microscope (Keyence, Osaka, Japan).

### Flow cytometry analysis of cells in the SFl

In 28 samples, cells of the SFl were viably frozen after aspiration. After thawing, the cells were stained with the fixable viability dye ViaKrome808 and subsequently fixed and permeabilized (PerFix-nc Kit, both Beckman Coulter). Cells were stained with the following antibodies: anti-human CD45-KrO (Beckman Coulter) as a general lymphocyte marker, anti-human CD68-PE, anti-human CD163-VioBlue (both Miltenyi Biotec). The CD45-staining was used to obtain the total amount of mononuclear cells in relation to macrophages in seroma fluid. Results were recorded with the CytoFlexLX cytometer and afterwards analyzed using Kaluza software (Kaluza Analysis 2.0, both Beckman Coulter) and gated accordingly (URL: https://www.beckman.de/flow-cytometry/software/kaluza). More information can be found in the supplement (Additional file 4, Additional Table 4).

### Detection of sCD163 in the patients’ sera at the time of the surgery

sCD163 in preoperatively sampled sera of patients was determined using a DuoSet ELISA kit (R&D Systems) according to manufacturer instructions as possible alternative prediction method for seroma formation. Samples were diluted at a 1:100 ratio with dilution buffer and the absorbance was measured at 450 nm with a plate reader (Tecan Spark). Results were evaluated by comparing the measured absorbance after blank subtraction to a standard curve provided in the kit.

### Statistical evaluation

SPSS Statistics (IBM, Version 29.0.1.0 (171)) and GraphPad Prism 10 were used for all statistical analyses. Two groups were formed as independent variables: cases with (seroma +) and without postoperative seroma formation (seroma-). The Shapiro–Wilk- and Kolmogorov–Smirnov-tests were used to categorize the distribution of the macrophage markers evaluated in this study. Since all parameters in this study were not normally distributed, the non-parametric Mann–Whitney-U-test was used. Pearson’s correlation coefficient was used for the metric (interval-scaled) data in this study. A p-value < 0.05 was considered statistically significant.

## Results

### Patient cohort description

In the SerMa pilot cohort, NST (non-specific type, 68 cases) and invasive lobular (16 cases) carcinomas occurred most frequently (Table 1). In 84 cases, the tumor was hormone receptor (HR) positive, 10 cases were HER2- positive, 10 cases were immunohistochemically triple-negative. The postoperative tumor size was mostly pT2 or lower (78 cases) and 32 cases showed pathological nodal involvement in the axilla. Out of the 53 cases who had received neoadjuvant chemotherapy, 16 achieved pathologic complete remission in the breast (ypT0). It has been shown previously that the axillary intervention and different tumor biological characteristics did not affect the patients’ risk of seroma development in this cohort ^9^.

**Table 1:** Patient and tumor characteristics.

	n(number)
Histopathological type	
NST	68
Invasive lobular	16
Mucinous	3
Papillary	3
Other	1
CIS	9
Hormone receptor status	
HR +	84
ER-/PR-	16
HER-2	
Positive (IHC + + + or FISH pos.)	10
Low (IHC + / + + or FISH neg.)	14
Negative (IHC 0)	67
Not determined^A^	9
Triple-negative status	10
Ki67	
< 20%	45
> 20%	46
Not determined^A^	9
Tumor size	
ypT0	16
pTis	8
pT1	30
pT2	24
pT3	17
pT4	5
Axillary nodal status	
pN0	64
pN1	20
pN2	10
pN3	2
pNx	4
Grading	
G1	13
G2	52
G3	33
No information available^B^	2

The percentages are identical to the absolute numbers. *NST* Non-special type, *CIS* Carcinoma in situ, *HR* Hormone receptor, *ER* Estrogen receptor. *PR* Progesterone receptor, *IHC* Immunohistochemistry, *FISH* Fluorescence in situ hybridisation. ^A^ in case of carcinoma in situ, ^B^ in case of external biopsy and diagnosing.

### Immunohistochemical staining and evaluation of the FFPE tissues

**Fig. 1:**
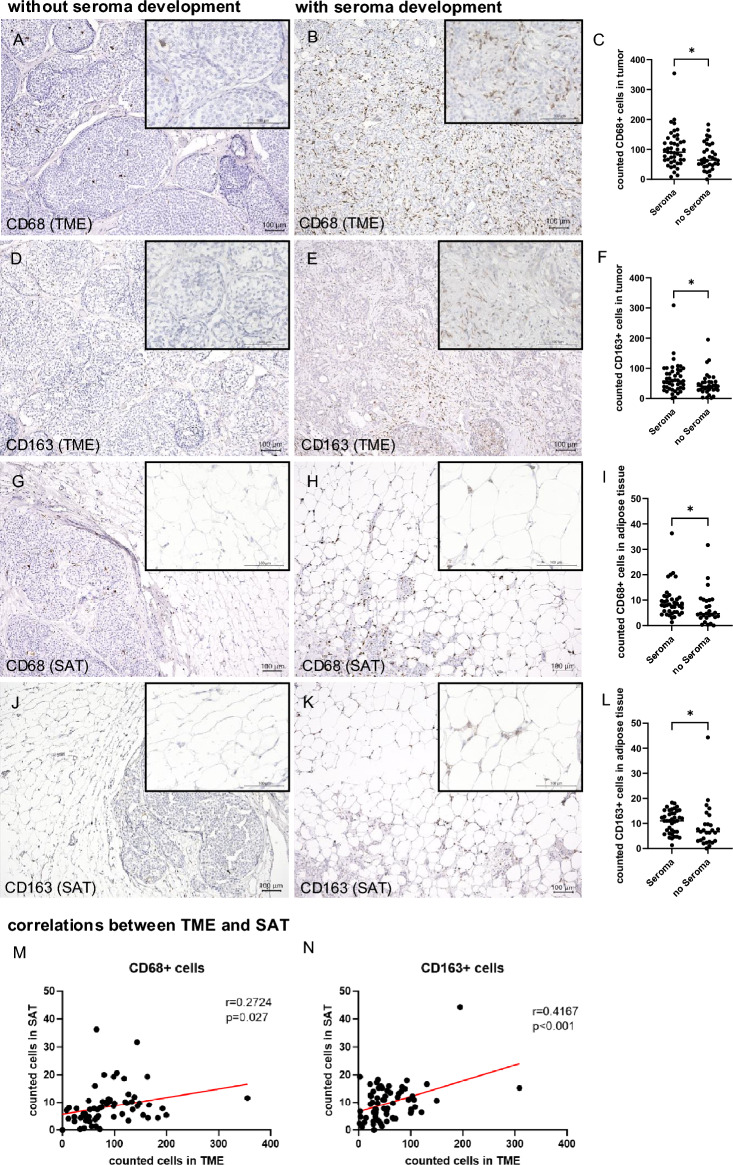
CD68 and CD163 expression in patients without and with seroma formation. CD68 expression in the TME of a patient without (**A**) and with (**B**) seroma development and differences illustrated within a dot plot (**C**). CD163 expression in the TME of a patient without (**D**) and with (**E**) seroma development and differences illustrated within a dot plot (**F**). CD68 expression in the SAT of a patient without (**G**) and with (**H**) seroma development and differences illustrated within a dot plot (**I**). CD163 expression in the SAT of a patient without (**J**) and with (**K**) seroma development and differences illustrated within a dot plot (**L**). Scale bars represent 100 μm. The dot plots visualize the distribution of positive cells in patients without and with seroma development. The horizontal lines represent the median values. *p < 0.05. Correlation analysis of macrophages (CD68 + cells) counted in the TME and SAT (**M**) and Correlation analysis of CD163 + cells counted in the TME and SAT (**N**).

The numbers of CD68 + (seroma + : median = 90.7cells, Interquartile Range (IQR) = 62.5–130.5; seroma-: median = 64.3 cells, IQR = 47.0–115-0, p = 0.036) and CD163 + cells (seroma + : median = 58.3, IQR = 33.0–91.4; seroma-: median = 40.7 cells, IQR = 28.3–55.3, p = 0.027) in the TME were significantly higher in cases with postoperative seroma formation compared to cases without (Fig. 1 A-F). In the SAT a significant increase was seen as well, nevertheless much less cells were counted (CD68 + cells (seroma + : median = 8.0 cells, IQR = 5.3–11.0; seroma-: median = 4.7 cells, IQR = 3.0–10.0, p = 0.013), CD163 + cells (seroma + : median = 11.0 cells, IQR = 6.7–15.0; seroma-: median = 6.7 cells, IQR = 3.0–9.7, p = 0.016)) (Fig. 1 F-L). There is a significant positive correlation between TME and SAT for CD68 + (Pearson correlation coefficient (r) = 0.272, p = 0.027) as well as CD163 + (r = 0.417, p < 0.001) cells (Fig. 1M,N).

### Triple immunofluorescence staining (CD68, CD163, PPARγ)

**Fig. 2:**
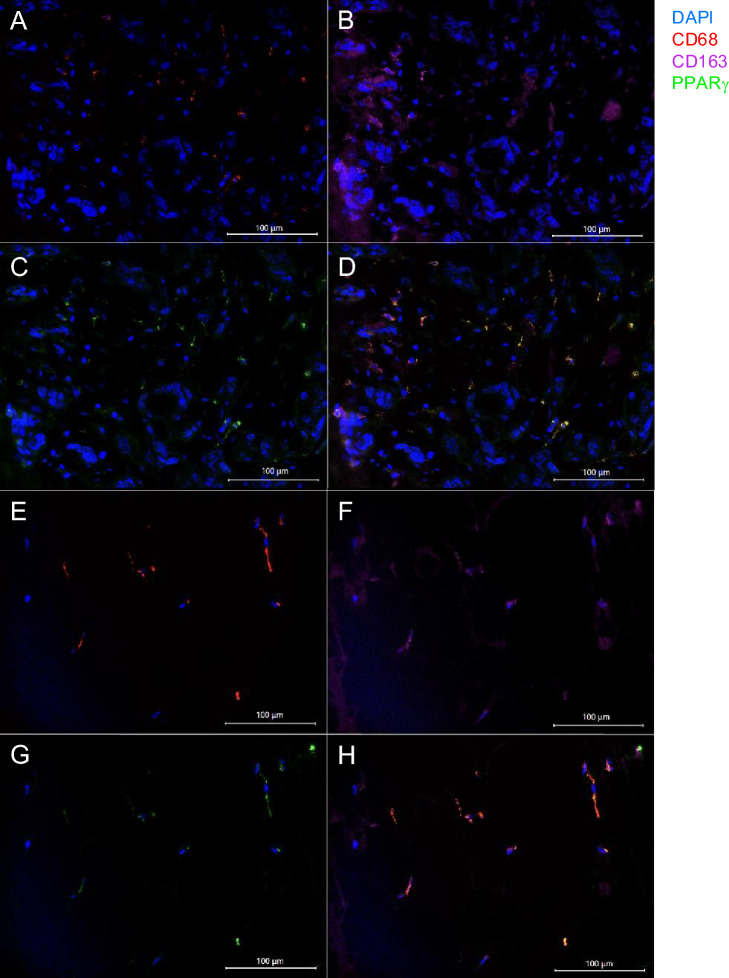
Immunofluorescence co-staining of CD68, CD163 and PPARγ. Immunofluorescence: Expression of CD68 in red (**A**), CD163 in violet (**B**), PPARγ in green (**C**) and the overlay (**D**) in the TME of a seroma-developer at a magnification of 40 × lens and expression of CD68 in red (**E**), CD163 in violet (**F**), PPARγ in green (**G**) and the overlay (**H**) in the SAT of the same patient at a magnification of 40 × lens. The scale bar represents 100 μm. Cell-nuclei of all eukaryotic cells are stained in blue by DAPI to verify the cellular identity.

Immunofluorescence analyses in FFPE slides showed the macrophage composition in both—TME and SAT—independent of postoperative seroma development. In the cases chosen for triple immunofluorescence staining of the TME, approximately half of the CD68 + macrophages (Fig. 2A) expressed CD163 (Fig. 2B). In the SAT, most CD68 + macrophages (Fig. 2E) also expressed CD163 (Fig. 2F). Nearly all of the macrophages expressed PPARγ (Fig. 2C,G). The triple-staining of all markers is shown in Fig. 2D and H.

### Flow cytometry analysis of cells in the SFl

**Fig. 3:**
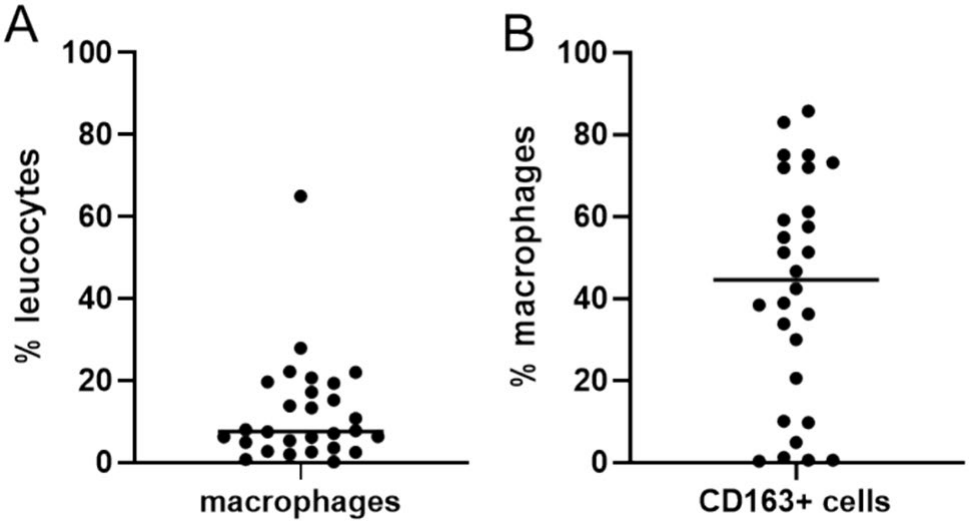
Cell composition of seroma fluid measured by flow cytometry. Percentage of macrophages (M$\varphi \text{, }\text{defined as CD68+ cells}$) within the leukocyte population (CD45 + cells) (**A**). The median percentage of leukocytes was 7.7% (IQR = 4.0–18.8%). Percentage of CD163 + cells within the macrophage population (**B**). The median amount of CD163 + cells was 44.6% (IQR = 12.7–69.3%). The horizontal lines represent median values.

The SFl cell content analyzed by flow cytometry showed high amounts of CD45 + cells (leukocytes) in the fluid. Of those leukocytes, a median of 7.7% (IQR = 4.0–18.8%) were macrophages (CD68 +) and a median of 44.6% (IQR = 12.7–69.3%) of the macrophages expressed CD163 (Fig. 3A,B). However, in contrast to the findings in the FFPE tissue, there is no significant correlation of cells measured in SFl and TME (CD68 + : r = − 0.248, p = 0.243; CD163 + : r = 0.128, p = 0.550) as well as SAT (CD68 + : r = − 0.190, p = 0.397; CD163 + : r = − 0.090, p = 0.690).

### Detection of serum(s)CD163 in the patients’ sera at the time of the surgery

**Fig. 4:**
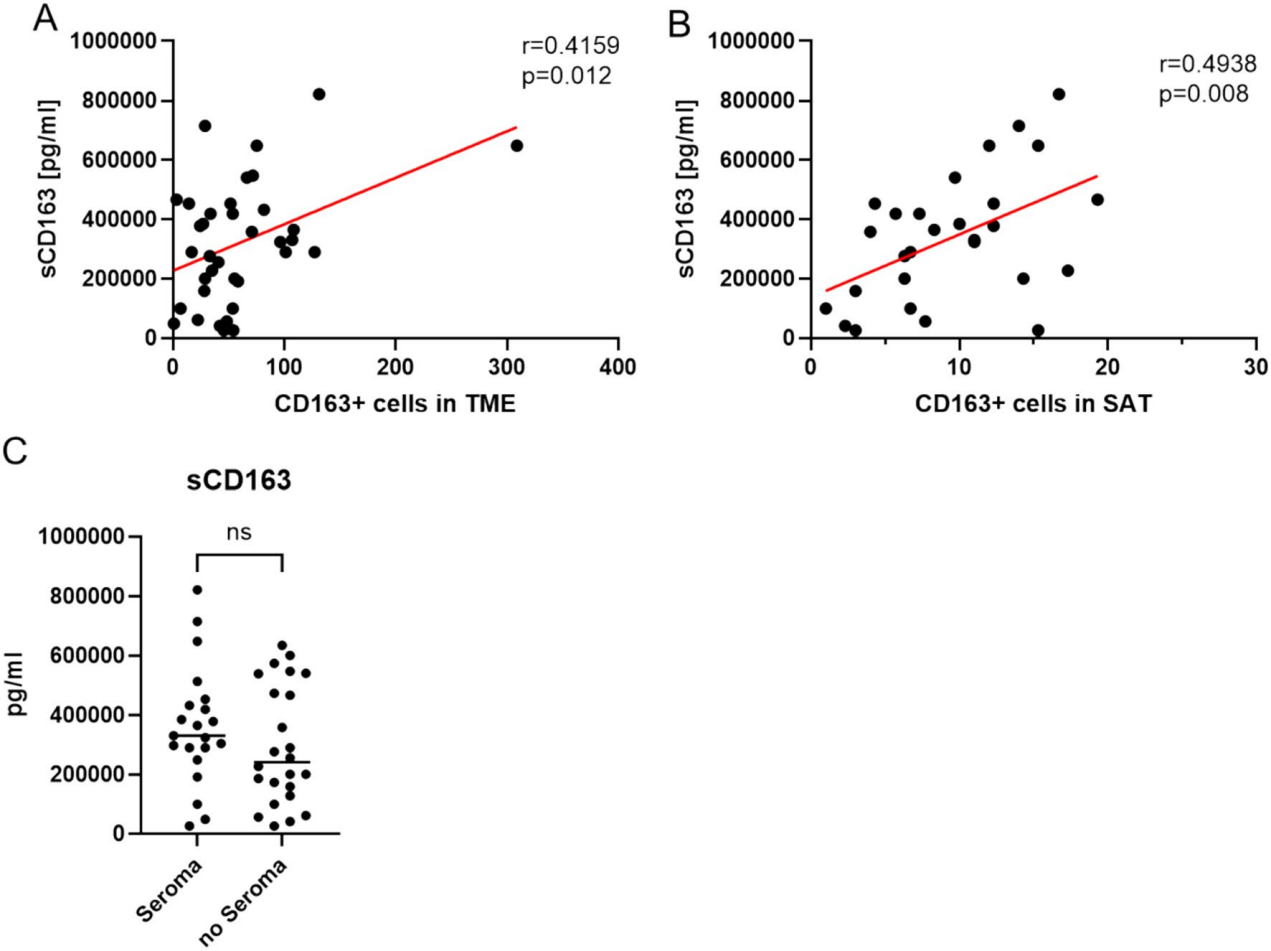
Correlation of the macrophage markers with sCD163 and its influence on seroma formation. Correlation analysis of sCD163 measured in the patients’ sera at time of surgery and CD163 + macrophages counted in the TME (**A**) and SAT (**B**). Mann–Whitney-U test of sCD163 levels measured in sera of patients at time of surgery with and without seroma formation (**C**). The horizontal line represents the median.

In contrast to CD163, CD 68 cannot be measured in serum. Therefore, all serum tests refer to CD163. In the Pearson’s correlation analysis, sCD163 correlated positively and significantly with CD163 + cells in the TME (r = 0.416, p = 0.012) and the SAT (r = 0.494, p = 0.008) (Fig. 4A,B). However, the difference between the levels of sCD163 of patients with and without seroma development did not reach significance (seroma + : median = 330.5 µg/ml, IQR = 269.1–442.5 µg/ml; seroma-: median = 241.1 µg/ml, IQR = 135.6–522.5 µg/ml, p = 0.251) (Fig. 4C).

## Discussion

The most important finding of this study is that the identification of an easily determinable immunohistochemical marker at the time of surgery is indicative of later seroma development.

Depending on their pathway of activation, TAM can be divided into two major subgroups: “classically activated”, pro-inflammatory M1 macrophages and tissue-repairing M2 macrophages, whose activation/ polarization pathway is induced by the cytokines IL4 and IL13 produced by T helper (Th) 2 cells ^16,23^. Previously, we could show a significant increase in Th2 and Th17 percentages in the SFl and blood of patients with seroma formation compared to the blood of healthy individuals ^12^. By confirming the presence of macrophages, including 44.6% CD163 + macrophages (M2) in the SFl, we have succeeded in further supporting the previous SerMa pilot study results ^9,12,13^. It needs to be further evaluated, if these macrophages are associated with the increase in Th (especially Th2 and Th17) cells found in SFl. The existence of macrophages in the SFl reinforces the theory that seroma formation is based on an immunological exudate rather than of lymphatic origin. Interestingly, there is no significant correlation between the macrophage populations in the TME and SAT and those measured in the SFl. Therefore, we hypothesize that the macrophage populations in the SFl reflect a tumor-independent macrophage polarization process. On the other hand, both CD68 + and CD163 + macrophages significantly correlate between TME and SAT, suggesting a dynamic exchange and migration between these tissues.

The second key-finding in this study is the different macrophage polarization of the TME compared to the SAT as we found that most CD68 + macrophages in the SAT were M2 macrophages (CD163 +) whereas merely half of the macrophages in the TME expressed CD163. PPARγ, which was expressed by most macrophages in our study, induces M2- differentiation by upregulating the fatty acid oxygenation pathway ^24^. Since fatty acids are naturally more abundant in the SAT than in the TME, it is reasonable to assume that the M2-polarization process takes place in the SAT and probably to a lesser extent in the TME. The PPARγ-induced pathway can be pharmacologically inhibited ^25^, making it an interesting target for translational applications to clinical research. For breast cancer patients in general, PPARγ has prognostic value; its cytoplasmatic expression is a negative prognosticator ^25^. Because PPARγ is also involved in insulin receptor functioning, it offers the possibility to consider treatments already in use for breast cancer related obesity ^26^, breast cancer progression ^27^, induction of apoptosis in breast cancer cells ^28^, inhibition of breast cancer growth through JAK2/STAT3 pathway ^29^, alleviating the effect of tamoxifen resistance by the compound I194496 and inhibiting the PPARγ/ACSL1/STAT3 signaling pathway in estrogen receptor-positive breast cancer ^30^ and enhancing cisplatin’s impact on triple-negative breast cancer by pioglitazone ^31^. Taken together, multiple lines of evidence indicate that activation/inhibition of PPARγ by natural or synthetic ligands exerts tumor modulating effects ^32^ and could therefore also be used to treat or prevent breast cancer surgery associated seroma development.

Although sCD163 was not predictive for seroma development, the M2 macrophage populations of the TME and SAT correlated positively with sCD163. The sCD163 is produced by proteolysis of membrane proteins and then released into serum ^33^. The results of the TME and SAT could lead to the development of a prognostic marker or marker combination regarding seroma formation, since according to our pilot study results, the solitary use of sCD163 for that purpose is currently not sufficient. The implementation and evaluation of the study results revealed several limitations. Due to the establishment of our methods, only 28 (70%) out of 40 punctured (100%) seroma cases were available for flow cytometry (Additional file 1). Moreover, CD68 + and CD163 + cells in the TME and SAT were counted manually instead of using a software-based tool since automated counting appeared less reliable due to the high density of cell nuclei. However, our evaluation was performed by two independent observers. We were also unable to statistically sufficiently investigate chemotherapy-induced changes in the TME and SAT, since in 16 cases the chemotherapy was so effective that ypT0 was achieved leading to a lack of cases in a possible “neoadjuvant-group”. Thus, possible changes in the TME and SAT induced by chemotherapy will be addressed in the bigger international SerMa study, improving the translational applicability of our findings due to better variable stratification. Moreover, we could not show a significant difference of sCD163 levels in preoperatively sampled serum between patients with and without postoperative seroma development in our study cohort. Nevertheless, in combination with other markers, a more precise prediction of seroma risk might be possible in future.

## Conclusions

Based on our results of the SerMa pilot study, the CD68 + and CD163 + macrophage infiltration of the TME and SAT is significantly associated with postoperative seroma development. In addition, the number of CD163 + macrophages in the SAT significantly correlates with sCD163, even though sCD163 was not a significant predictor for seroma development. These results will be verified in the multicentric, prospective SerMa study. Our results may be the basis for the development of a prognostic marker combination predicting seroma risk in patients undergoing breast cancer surgery with or without reconstruction.

## Supplementary Information

Supplementary Information.

## Data Availability

The datasets used and analyzed during the current study are available from the corresponding author on reasonable request.

## Abbreviations

SFl: Seroma fluid
TAM: Tumor associated macrophages
TME: Tumor microenvironment
SAT: Surrounding adipose tissue
PPAR-γ: Peroxisome proliferator-activated receptor gamma
sCD163: Soluble CD163
FFPE: Formalin-fixed paraffin embedded
DCIS: Ductal carcinoma in situ
CIS: Carcinoma in situ
SLNB: Sentinel lymph node biopsy
ALND: Axillary lymph node dissection
TAD: Targeted axillary lymph node dissection
DAPI: 4′,6-diamidino-2-phenylindole
NST: Non-specific type
Th: T helper
