# Supplementary material for: Macrophage polarization is associated with postoperative seroma development in breast cancer in the SerMa pilot cohort
Source: Sci Rep. 2025 Sep 12;15:32442. doi: 10.1038/s41598-025-17139-2 (PMC12432247; doi:10.1038/s41598-025-17139-2)
Supplement: Supplementary file 1 — Supplementary Information. [file 41598_2025_17139_MOESM1_ESM.docx]

**Supporting information**

**Contents:**

Additional file 1: Number of cases eligible for sufficient final evaluation and reasons for drop-out

Including: Additional table 1: Number of eligible cases for sufficient final evaluation

Additional file 2: Staining protocols and antibodies used for immunohistochemistry

Including: Additional table 2: Antibodies used for immunohistochemical antibody staining

Additional file 3: Staining protocol and antibodies used for immunofluorescence

Including: Additional table 3: Primary (p) and secondary (s) antibodies used for immunofluorescence staining

Additional file 4: Protocol of flow cytometry- based analyses of the cells within the seroma fluid

Including: Additional table 4: Antibodies used for flow cytometry staining

**Additional file 1: Number of cases eligible for sufficient final evaluation and reasons for drop-out**

**Additional table 1: Number of eligible cases for sufficient final evaluation**

| Staining | Total cases | Loss due to ypT0 | Losses due to logistical reasons | Losses due to the lack of fat tissue | Eligible for final evaluation |
| --- | --- | --- | --- | --- | --- |
| CD68 and CD163 (TME) | 100 | 16 | 4 | - | **80** |
| CD68 and CD163 (SFT) | 100 | 16 | 4 | 14 | **66** |

Statement regarding the analysis of sCD163 in the patients’ sera at the time of surgery: Patients could choose to give a blood sample for this purpose. 49 out of the 100 cases agreed. We therefore had 49 cases for this part of the study.

Statement regarding the flow cytometric analysis of the seroma fluid: Seroma fluid of the first 12 cases was used for establishing our method. Therefore, only the remaining 28 out of 40 punctured seromas were available for the final flow cytometry evaluation.

**Additional File 2: Staining protocols and antibodies used for immunohistochemistry**

For immunohistochemical antibody staining, the samples were deparaffinated in a Xylol-like solution (Roticlear®) for 20 Minutes and afterwards rinsed with 100% ethanol. To inhibit endogenous peroxidase activity, the specimen were bathed in a solution of Methanol and 3% H_2_O_2_ for 20 Minutes. Afterwards, the slides were rehydrated in distilled water using a descending alcohol line before cooking them in a sodium-citrate buffering solution (pH=6.0), unmasking epitopes. They were then rinsed with distilled water and washed for 2x2 minutes in phosphate buffered saline (PBS). Subsequently, the samples were treated for 5 minutes using a blocking solution (Reagent 1, Zytochem Plus HRP Polymer System (Mouse/ Rabbit) by Zytomed) and incubated with the Anti-CD68 or Anti-CD163 antibody for 16h at a temperature of 4 °C (39.2°F). Following, the specimen were washed in PBS and treated with a post-block solution for 20 minutes (Reagent 2, Zytochem Plus HRP Polymer System (Mouse/ Rabbit) by Zytomed). After again being washed in PBS, the slides were incubated in horse-radish peroxidase (HRP) -polymer (Reagent 3, Zytochem Plus HRP Polymer System (Mouse/ Rabbit) by Zytomed) and washed in PBS. Subsequently, diaminobenzidine (DAB) solution (Liquid DAB+ Substrate Chromogen System by Agilent) was applied to each slide for 30 seconds for substrate coloring. To stop the coloring reaction, the samples were washed with distilled water immediately. In the following step, the specimen were counterstained for 2 minutes using Mayer ’s acid hemalum and stained blue for 5 min in tap water. After dehydration in an ascending alcohol line, the slides were treated with Roticlear® and cover slipped with RotiMount® (Carl Roth, Germany). A human tonsil (CD68) and placenta (CD163) were also stained as positive controls.

**Additional table 3:** **Antibodies used for immunohistochemical antibody staining**

| Antibody | Isotype | Clone | Dilution | Source |
| --- | --- | --- | --- | --- |
| Anti-CD68 | Rabbit IgG | Monoclonal, clone D4B9C | 1: 1000 | Cell Signaling |
| Anti-CD163 | Mouse IgG1 | Monoclonal, clone OTI2G12 | 1: 2000 | Abcam |

**Additional file 3: Staining protocol and antibodies used for immunofluorescence**

For immunofluorescence staining, the sections were first deparaffinated in a Xylol-like solution (Roticlear®) for 20 Minutes and then rehydrated using a descending alcohol line. To unmask epitopes, the specimen were cooked in a sodium-citrate buffering solution (pH=6.0) and afterwards rinsed with distilled water and washed in PBS for 2x2 minutes. Following, the samples were treated with immunofluorescence blocking buffer (Cell Signaling, Nr. 12411 S) for 60 minutes at room temperature, blocking unspecific binding sites. Subsequently, the slides were incubated with primary Anti-CD68 and Anti-CD163 antibodies for 16h at a temperature of 4°C (39.2°F). The samples were then washed with PBS and incubated with a combination of Cy-5- conjugated Goat-Anti-Mouse secondary antibodies, resulting in violet coloration of CD163+ antigens, and Cy-3- conjugated Goat-Anti-Rabbit secondary antibodies, resulting in a red coloration of CD68+ antigens, at room temperature for 30 minutes in the dark. Afterwards, the specimens were washed in PBS and free binding sites of the Goat-Anti-Mouse secondary antibodies were blocked using Fab Fragment Donkey Anti-Goat IgG (H+L) (Dianova, Dilution 1:50). Subsequently, the slides were washed in PBS and the Anti- PPARγ primary antibodies were applied for 1h at room temperature. After being washed in PBS, the slides were treated for 30 minutes at room temperature in the dark with Alexa Flour 488 conjugated Goat-Anti-Rabbit secondary antibodies, resulting in green coloration of PPARγ+ antigens. Subsequently, the samples were again washed in PBS for 4 minutes and afterwards treated with TrueBlack (dilution: 1:20 in 70% ethanol) for 1 minute at room temperature in the dark. After another PBS washing step, the specimens were dried at room temperature and mounted with DAPI (4′,6-diamidino-2-phenylindole) containing mounting medium, resulting in blue staining of the cell nuclei.

**Additional table 3: Primary (p) and secondary (s) antibodies used for immunofluorescence staining**

| Antibody | Isotype | Clone (p)/ conjugation (s) | Dilution | Source |
| --- | --- | --- | --- | --- |
| Anti-CD68 (p) | Rabbit IgG | Monoclonal, clone D4B9C | 1: 1000 | Cell Signaling |
| Anti-CD163 (p) | Mouse IgG1 | Monoclonal, clone OTI2G12 | 1: 2000 | Abcam |
| Anti-PPARγ (p) | Rabbit IgG | Polyclonal | 1:100 | Abcam, cat. no. ab59256 |
| Goat-Anti-Mouse (s) | IgG (H+L) | Cy5 conjugated (violet) | 1:100 | Dianova, cat. no. 115-175-166 |
| Goat-Anti-Rabbit (s) | IgG (H+L) | Cy3 conjugated (red) | 1:500 | Dianova, cat. no. 111-165-003 |
| Goat-Anti-Rabbit (s) | IgG (H+L) | Alexa Flour 488 conjugated (green) | 1:500 | Thermo Fisher, cat. no. A11008 |

**Additional file 4: Protocol of flow cytometry- based analyses of the cells within the seroma fluid**

After centrifugation of the seroma fluid, the supernatant was removed and cells were counted using a haemato-counter (Sysmex XP300; SYSMEX, Norderstedt, Germany). Cells were viably frozen in 1 × 10^7 aliquots and stored in liquid nitrogen. After thawing, the cells were stained with the fixable viability dye ViaKrome 808 (Beckman Coulter) for 20 minutes at room temperature. After washing with PBS, the cells were fixed and permeabilized using the PerFix-nc Kit (Beckman Coulter) according to manufacturer’s instructions and subsequently stained with antibodies labeled with immune fluorescent dyes (eTable3). Stained cells were washed with PBS before recording using the flow cytometer CytoFlex LX (Beckman Coulter). Single color staining was included for compensation purposes. We made sure to record an appropriate number of cells (50,000 lymphocytes).

**Additional table 4:** **Antibodies used for flow cytometry staining**

| Antibody | Label | Clone | Dilution | Source |
| --- | --- | --- | --- | --- |
| anti-human CD68 | PE | Y1/82A | 2µl/test | Miltenyi Biotec |
| anti-human CD163 | VioBlue® | REA812 | 2µl/test | Miltenyi Biotec |
| anti-human CD45 | Krome Orange | J33 | 5µl/test | Beckman Coulter |
